# Authors’ reply

**Published:** 2010

**Authors:** P. Adithya Malolan, Vishak Acharya, B. Unnikrishnan

**Affiliations:** *Department of Chest Diseases, Kasturba Medical College, Mangalore, India. E-mail: amalolanp@gmail.com*; 1 *Department of Chest Diseases, Kasturba Medical College, Mangalore, India.*; 2 *Department of Community Medicine, Kasturba Medical College, Mangalore, India.*

Sir,

The authors would like to thank Lung India for their excellent support. In response to the above letter,[1] the authors would like to thank you for such an inspiring review and would also like to add that the study ‘FEV_6_ as screening tool in spirometric diagnosis of obstructive airway disease’[2] was only a pilot study, conducted mainly to check for the feasibility of carrying out such a study in an Indian setting. Currently, planning is under way for a similar study on a larger scale with a larger sample size including normal subjects also, which will enable us to generalize the results that we found in our original study.
